# Evaluation of Episodic and Lexical Metamemory and Executive Function in Healthy Older Adults

**DOI:** 10.1093/geroni/igab046.2623

**Published:** 2021-12-17

**Authors:** Atsuko Hayashi

**Affiliations:** Kobe University, Kobe, Hyogo, Japan

## Abstract

In older adults, it is important to maintain awareness of memory as well as memory performance. However, it is not clear whether the awareness of episodic and lexical memory changes with age and is related to self-evaluation of memory and executive function. Here age-related changes and the relationship between metamemory, executive function, and metamemory scale were investigated. Healthy old (n=40) and young (n=34) groups participated in this study. In the episodic memory task, participants were asked to memorize ten Kanji words and to estimate the number of words they could recall after ten minutes. In the lexical memory task, they rated the likelihood that they could write a target Kanji word written in hiragana and then wrote them down. They were also asked to complete the metamemory in adulthood(MIA) and the position stroop task. In the episodic and lexical memory and the position stroop task and MIA subscales, the performances of the younger group were significantly better than those of the older group. In the episodic memory task, there were correlations between the metamemory and MIA subscales in both groups, but in the lexical memory task, only in the old group. No correlation was found between the results of both memory tasks and the stroop test. These results suggest that older people overestimate memory performances in the episodic and lexical memory tasks and metamemory performances may be associated with self-evaluation of memory. In addition, metamemory might not be related to frontal lobe function as shown in executive function tasks.

